# A multicenter prospective study of 515 febrile neutropenia episodes in Argentina during a 5-year period

**DOI:** 10.1371/journal.pone.0224299

**Published:** 2019-10-31

**Authors:** Roberto L. Parodi, Mariana Lagrutta, Mauro Tortolo, Estefanía Navall, María S. Rodríguez, Gervasio F. Sasia, Lucas F. De Candia, Matias A. Gruvman, Oscar Bottasso, Alcides A. Greca

**Affiliations:** 1 1º Cátedra de Clínica Médica y Terapéutica, Facultad de Ciencias Médicas, Universidad Nacional de Rosario, Rosario, Santa Fe, Argentina; 2 Servicio de Clínica Médica, Hospital Provincial del Centenario, Rosario, Santa Fe, Argentina; 3 Servicio de Clínica Médica, Hospital Provincial de Rosario, Rosario, Santa Fe, Argentina; 4 Instituto de Inmunología Clínica y Experimental de Rosario, Universidad Nacional de Rosario, Consejo Nacional de Investigaciones Científicas y Técnicas, Rosario, Santa Fe, Argentina; Consejo Nacional de Investigaciones Cientificas y Tecnicas, ARGENTINA

## Abstract

For better management of patients with febrile neutropenia, our study investigated the epidemiologic, microbiologic, and clinical characteristics of adult inpatients with febrile neutropenia and their mortality-associated factors. To this end, we carried out a prospective, observational, multicenter study in 28 Argentinian hospitals between 2007 and 2012. We included 515 episodes of febrile neutropenia from 346 patients, median age 49 years. Neutropenia followed chemotherapy in 77% of cases, half of the cases due to hematological malignancies. Most episodes were classified as high-risk according to MASCC criteria, and 53.6% of patients were already hospitalized at the onset of febrile neutropenia. Bloodstream infections were detected in 14% episodes; whereas an infectious source of fever was identified in 80% of cases. Mortality rate achieved to 14.95%. The binary regression analysis showed that persistence of fever at day 7, or neutropenia at day 14, dehydration and tachycardia at the onset of febrile neutropenia as well as prior infections were significantly associated with mortality. In addition to expanding our current knowledge on the features of adult patients with febrile neutropenia, present findings provide useful information for better management of them in Argentina, given the appropriate representativeness of centers participating in the study.

## Introduction

Febrile neutropenia constitutes a differentiated clinical entity that is becoming increasingly frequent in daily hospital practice. The leading cause of neutropenia results from chemotherapy in patients with malignancies, making them more susceptible to bacterial infections because of the reduction of this critical component from innate defense mechanisms [1, 2]. In turn, chemotherapy damages the integrity of the gastrointestinal mucosa, favoring the invasion of endogenous germs [1, 3–6]. Since the ability to develop an adequate inflammatory response is impaired, the spectrum of clinical manifestations allowing to suspect an infectious process is also compromised, with fever being frequently the sole indicator of that [1, 5, 7, 8]. The progression of infection in neutropenic patients may be fast, for which the early administration of empirical treatment is crucial, even before confirming any infectious source [1, 5, 8].

Current advances in terms of disease physiopathology, new imaging-based diagnosis, as well as microbiological and serological tests, resulted in better control measures and decreased morbimortality [5]. Nevertheless, case numbers continue to be significant, making the issue still relevant in terms of a health problem [1, 9, 10]. On the other hand, changes in the profile of the etiological agents together with the growing antimicrobial resistance renders the situation even more complex [5, 6, 8, 10].

It follows that the management of febrile neutropenic patients remains challenging [1, 5, 10]. International and regional guidelines developed for the management of these patients, also establish the need for a proper epidemiological knowledge from different regional scenarios [5, 6, 8, 10].

Given this background, we sought to identify the epidemiological, microbiological, clinical, and therapeutic characteristics of patients with febrile neutropenia from centers mostly located in the central area of Argentina. In a second step, we also searched for factors likely to be associated with fatal outcome.

## Materials and methods

### Participants

All participants provided written and informed consent for the study, which was approved by the Ethic Committee from the School of Medical Sciencies National University of Rosario on July 2007 (resolution # 326/2007) in accordance with the World Medical Association’s Declaration of Helsinki.

The study consisted of a multicenter, observational and prospective study of febrile neutropenia episodes in adult inpatients from 01-Aug-2007 to 02-Mar-2012, in 28 Hospital Departments of Internal Medicine from Argentina, led by investigators from “Hospital Provincial del Centenario” University Hospital. All participating centers habitually treat patients with febrile neutropenia episodes (FNEs) according to periodically revised local protocols, following national and international guidelines and recommendations. Inclusion criteria comprised age ≥15 years, along with the presence of neutropenia and fever according to the following definitions: for neutropenia a neutrophil count below 500 / mm^3^; or less than 1,000 / mm^3^ and likely to decrease to less than 500 / mm^3^ in the next 48 hours; in the case of fever, presence of a single record of axillary temperature > 38.3° C, or ≥ 38° C for one hour or more.

Day 0 corresponded to the time when the patient fulfilled inclusion criteria and was included in the study. The existence of an infective source was established provided the clinician made the diagnosis of a clinically documented infection based on clinical and imaging data. A microbiological infection was confirmed, whenever a microorganism was isolated in cultured samples from a clinically suspected source.

Criteria for study completion comprised the remission of fever and neutropenia. Information on whether the patient remained in the hospital for other reason or had been released from it was recorded at that time point. Death of the patient or the referral to another care center was also regarded as end of study.

For the remission of fever, we considered values lower than 38°C for the latest 48 hours; whereas values higher than 500 neutrophils / mm^3^ for at least during the two preceding days were regarded as neutrophil recovery.

### Procedures

Evaluation of patients consisted of a complete physical examination, biochemistry and hematology studies, sets of blood cultures, or additional samples from infected sites if needed. Recorded data included demographic and epidemiologic variables, cause of neutropenia, i.e., cancer chemotherapy, underlying disease, and comorbidities. Information on patient antecedents before day 0 included: ambulatory or hospitalized condition at the onset of FNEs, prior use of antibiotics, presence of previous fungal infection, or neutropenia episodes. Data from physical examination, routine laboratory tests on admission, blood and urine cultures, use of a central venous catheter (CVC), empiric antibiotic treatment, administration of granulocyte colony-stimulating factor (G-CSF), detection of clinical or microbiological infection, evolution and outcome (death, recovery or referral) were also recorded. The clinical source of fever was registered by the medical staff according to its location: lung, skin, gastrointestinal tract, urinary, and central nervous system, among others. The biochemistry and microbiological studies were carried out at each center according to standard procedures.

The MASCC risk index (Multinational Association for Supportive Care in Cancer) [11] was estimated on admission, as did the ECOG (Eastern Cooperative Oncology Group) performance score [12, 13]. The MASCC risk index developed and validated to identify febrile neutropenic patients with different risk of complications and death [8, 14–16], served to separate individuals into low or high-risk groups (values ≥ or < 21 points, respectively) [11].

The ECOG scale, which is widely used to assess the functional status of cancer patients, mainly their daily life ability and autonomy, ranged from 0 (patients with autonomy and normal activity) to 4 (bed-ridden patients at high risk of complications during febrile neutropenia) [13, 17].

As stated, all patients or their legal representatives provided written informed consent before enrolment in the study.

### Statistical analysis

Quantitative results were expressed as median and 25–75 percentiles when required. Univariate analysis was performed by using the Chi-square test or Fisher's exact test for categorical variables, whereas the Mann-Whitney test was employed for continuous variables (survivors versus non-survivors comparisons). Variables yielding significant differences in univariate analysis were entered into a binary logistic regression model for the multivariate approach. The level of statistical significance was set at p <0.05. Data were analyzed by employing the SPSS package.

## Results

### Baseline characteristics of the sample population

A total of 346 patients, experiencing 515 FNEs were enrolled. Age at the FNE episode was 49 (34–60) years, distributed among 227 women and 288 men. Nearly half of episodes (46%) occurred in the range age of 40 to 64 years. Data about patient characteristics on study admission (day 0) are depicted in Table 1. As shown, 53.6% of patients were already hospitalized at the onset of the episode, whereas one-third of them had the antecedent of prior FNEs or were undergoing antibiotic treatment before developing neutropenia. Among those receiving prophylaxis (n = 63, 12.2%), regimens included cotrimoxazole (n = 37, 7.2%), quinolones (n = 19, 3.7)%, or other compounds (n = 7, 1.3%).

**Table 1:** Characteristics of patients at the beginning of 515 of febrile neutropenia episodes.

**Sex**	Men		288 (55.9%)
	Women		227 (44.1%)
**Age (years)**			49 (34–60)
**Comorbidities**	HIV		51 (9.9%)
	Immunosuppressants		49 (9.5%)
	Glucocorticoid therapy		28 (5.4%)
	Diabetes		28 (5.4%)
	COP		22 (4.3%)
	CHF		15 (2.9%)
	CRI		15 (2.9%)
	Hemodialysis		6 (1.2%)
**Other antecedents**	Radiotherapy		39 (7.6%)
	Bone marrow transplantation		18 (3.5%)
	Previous episodes of neutropenia		167 (32.4%)
	Central venous catheter at the onset of FNE		98 (19%)
	Previous fungal invasive infection		27 (5.2%)
	Hospitalized at the onset of FNE		276 (53.6%)
	Prior use of antimicrobials		161 (31,3%)
		Bacterial prophylaxis	63 (12.2%)
		Prior infection at the onset of FNE	98 (19%)
	No antibiotics		354 (68.7%)
**Physical examination at the onset of FNE**	Hypotension		85 (16.5%)
	Tachycardia		179 (34.8%)
	Dehydration		64 (12.4%)
	Tachypnea		152 (29.5%)
	Oliguria		28 (5.4%)
**ECOG score (n = 472)**^**1**^	ECOG 0		89 (18.9%)
	ECOG 1		159 (33.7%)
	ECOG 2		95 (20.1%)
	ECOG 3		93 (19.7%)
	ECOG 4		36 (7.6%)
**MASCC risk index at the onset of FNE**	High risk <21		491 (95.3%)
	Low risk ≥21		24 (4.7%)
**Laboratory at the onset of FNE**	Leukocytes (cells/mm3)		800 (400–1400)
	Neutrophils (cells/mm3)		100 (0–348)
	Deep Neutropenia (<100 cells/mm3)		242 cases (47%)
	Hemoglobin (g/dl)		8,9 (7,4–10.4)
	Platelets (cells/mm3)		60000 (18500–120000)

Quantitative values are represented as median (25–75 percentiles). Percentages are indicated in parentheses COP: chronic obstructive pulmonary disease; CHF: congestive heart failure; CRI: chronic renal insufficiency. ECOG: Eastern Cooperative Oncology Group; FNE: febrile neutropenia episode; MASCC: Multinational Association for Supportive Care in Cancer.

^1^The ECOG score was calculated in 472 episodes.

The main comorbidities were HIV infection, treatment with immunosuppressants or corticosteroids, and diabetes. The MASCC risk index corresponded to the high-risk group (<21) in 95.3% of cases, while 47.4% of patients presented a ≥2 ECOG score.

The median neutrophil counts by total episodes was 100 (0–348) cells/mm^3^, with 242 FNEs (47%) showing profound neutropenia (≤100 neutrophils / mm3). Tachycardia and tachypnea, followed by hypotension, dehydration, and oliguria were the most frequent alterations seen on physical examination at the onset of FNEs (Table 1).

Most FNEs (395) were secondary to chemotherapy, 291 of them due to oncohematological malignancies, i.e., non-Hodgkin's lymphoma (NHL), acute myeloid leukemia (AML), acute lymphocytic leukemia (ALL), and Hodgkin lymphoma. Another 104 episodes (20.2%) resulting from chemotherapy, had a diagnosis of solid tumors, such as breast, lung, and colorectal cancer, in decreasing order. In 120 episodes (23.3%), neutropenia was unrelated to chemotherapy, and due to the presence of primary hematological disorders, infections, drugs or autoimmune diseases. In 14 cases, neutropenia causes remained unidentified (Table 2).

**Table 2:** Causes of neutropenia in the present series of febrile neutropenia episodes.

Cause	N
**POSTCHEMOTHERAPY–ONCOHEMATOLOGIC MALIGNANCIES (n:291; 56.5%)**	
Non-Hodgkin Lymphoma	107 (36.8%)
Acute Myeloid leukemia	94 (32.3%)
Acute lymphoblastic Leukemia	43 (14.8%)
Hodgkin disease	17 (5.8%)
Chronic lymphocytic Leukemia	15 (5.2%)
Multiple Myeloma	7 (2.4%)
Others	8 (2.7%)
**POSTCHEMOTHERAPY–SOLID TUMORS (n:104; 20.2%)**	
Brest	21 (20.2%)
Lung	19 (18.3%)
Colorectal	12 (11.5%)
Cervix	11 (10.6%)
Testicle	8 (7.7%)
Oral cavity	7 (6.7%)
Sarcoma	6 (5.8%)
Ovary	5 (4.8%)
Others	15 (14.4%)
**NEUTROPENIA UNRELATED TO CHEMOTHERAPY (n:106; 20.6%)**	
**Hematological**	**54 (50.9%)**
• Acute Myeloid leukemia	22
• Myelodysplastic syndrome	10
• Aplastic anemia (pancytopenia)	9
• Lymphoproliferative disorders	4
• Tricholeukemia	4
• Acute lymphoblastic Leukemia	3
• Other hematologic causes	2
**Infections**	**28 (26.4%)**
• HIV	25
• Histoplasmosis	2
• Dengue	1
**Drugs**	**13 (12.3%)**
• Methimazole	5
• Methotrexate	3
• Other drugs	5
**Others**	**11 (10.4%)**
• Hypersplenism	4
• Autoimmune diseases	4
• Bone marrow metastasis	2
• Primary humoral immunodeficiency	1
**NEUTROPENIA UNRELATED TO CHEMOTHERAPY, UNKNOWN CAUSES (n = 14; 2.7%)**	

Blood cultures performed on day 0 were positive in 72 of FNEs (14%), showing a slight predominance of Gram-negative bacilli (52.8%). The main isolated microorganism was *Escherichia coli*, followed by methicillin-sensitive *Staphylococcus aureus*, *Klebsiella pneumoniae*, and pseudomonas species (Table 3).

**Table 3:** Results from cultures performed at day 0 and during the evolution of febrile neutropenia episodes.

Blood cultures	Isolated microbe (n)	*n*
Blood cultures at the onset (n = 515)	**Gram-negative bacilli**	**38**
	*Escherichia coli (18); Klebsiella pneumonia (7); Pseudomonas sp (7); Acinetobacter baumannii (1); Other Gram-negative bacilli (5)*	
	**Gram-positive cocci**	**34**
	*Methicillin-susceptible Staphylococcus aureus (15); Methicillin-resistant Staphylococcus aureus (5); Coagulase-negative staphylococci* (4); *Streptococcus viridans (4); Streptococcus pneumonia (3); Enterococcus sp*. *(2); Streptococcus pyogenes (1)*	
	**Total**	**72**
Blood cultures during the evolution (n = 312)	Isolated microbe (n)	
	**Gram-negative bacilli**	**18**
	*Klebsiella pneumoniae (7)*, *Escherichia coli (4)*, *Acinetobacter baumannii (3)*, *Pseudomonas sp (2)*, *Stenotrophomonas maltophilia (1)*, *Klebsiella oxytoca (1)*	
	**Gram-positive cocci**	**15**
	*Staphylococcus coagulase negative (4)*, *Methicillin-susceptible Staphylococcus aureus (5)*, *Streptococcus viridans (2)*, *Enterococcus faecium (2)*, *Enterococcus faecalis (1)*, *Methicillin-resistant Staphylococcus aureus (1)*	
	**Total**	**33**
**Urine culture**	Isolated microbe (n)	
Urine cultures at the onset (n = 515)	**Gram-negative bacilli**	**40**
	*Escherichia coli (31)*, *Klebsiella pneumonia (4)*, *Enterobacter cloacae(2)*, *Acinetobacter baumannii (1)*, *Proteus mirabilis (1)*, *Pseudomonas sp (1)*	
	**Gram-positive cocci**	**5**
	*Enterococcus faecalis (2)*, *Coagulase-negative staphylococci* (1), *Streptococcus viridans (1)*, *Methicillin-susceptible Staphylococcus aureus (1)*	
	**Yeast**	**3**
	Candida (1)	
	Other yeast (2)	
	**Polymicrobial growth**	**1**
	**Total**	**49**
Urine cultures during evolution (n = 128)	**Isolated microbe** (n)	
	**Gram-negative bacilli**	**10**
	*Escherichia coli (7)*, *Serratia marcescens (2)*, *Klebsiella pneumoniae (1)*	
	**Yeast**	**3**
	Candida (3)	
	**Total**	**13**
Stoll cultures at the onset (n = 103)	**Gram- negative bacilli (n)**	**1**
	*Campylobacter sp*.(1)	
	**Parasitosis**	**2**
	*Cryptosporidium neoformans* (1), *Blastocystis hominis* (1)	
	**Total**	**3**
Stoll cultures during evolution (n = 22)	**None**	

Microorganisms were indicated according to decreasing order of presentation

When analyzing the association between antimicrobial prophylaxis and the type of bacteria recovered from blood cultures, we found an insignificant trend for Gram-positive predominance among patients undergoing prophylaxis (5/8 Gram-positive bacteremias from patients under prophylaxis, versus 29/64 from those with no prophylaxis, p = 0.463). The same trend was recorded when analyzing prophylaxis according to cotrimoxazole and quinolones treatments (data not shown).

Throughout the evolution of FNEs, one to three additional blood cultures were taken in 312 cases, 33 of them yielding positive results (10.6%). Again, there was a small predominance of Gram-negative bacilli, with *K*. *pneumoniae* being the most frequently isolated one (Table 3). Urine cultures performed in all FNEs yielded positive results in 49 cases (9.5%); as did another 13 cases during their evolution. In both occasions, *E*. *coli* predominated (Table 3).

Mycological blood cultures performed at day 0 (n = 153) were all negatives, whereas in another 90 samples collected during evolution, the presence of *Candida albicans* was found in 2 out of them (2.2%).

Stoll cultures were carried out as required. Among 103 stoll cultures performed at the onset of FNE, 3 of them led to pathogen isolation, which may be incriminated as being possibly involved in diahrrea (Table 3).

### Confirmed infection

In overall, an infectious cause was identified in 80% of FNEs. More than 68% of FNEs (n = 355) had a diagnosis of an infectious process, based on clinical data (n = 235, 46%) or clinical and microbiological evidence (n = 120, 23%). There were another 58 cases (11%) with a positive microbiological result but no clinical focus. In the remaining 102 episodes, clinical or microbiological evidence of infection was lacking.

The distribution of clinical infections at day 0 was as follows: pneumonia (n = 96, 18.6%), gastrointestinal tract -mostly diarrhea- (n = 84, 16.3%), skin and soft tissues (n = 53, 10.3%), pharynx/oral cavity (n = 49, 9.5%), severe mucositis-related infections (n = 24, 4.6%), perianal zone (n = 20, 3.9%), phlebitis (n = 19, 3.7%), CVC-associated infection (n = 9, 1.7%), and other tissues (n = 8, 1.5%). Some patients presented more than one clinical infection,exhibiting a similar trend of site distribution, that is pneumonia as the predominant infection throughout the episode course (n = 31, 6%), followed by affectation of the gastrointestinal tract (n = 20, 3.9%), skin and soft tissues (n = 18, 3.5%), pharynx/oral cavity (n = 14, 2.7%) phlebitis (n = 6, 1.2%), perianal zone (n = 6, 1.2%), severe mucositis-related infections (n = 5, 1%), and a case of CVC-associated infection.

### Treatment

Two third of FNEs were given G-CSF (n = 341). Information dealing with empiric antibiotic therapy on day 0 is provided in Table 4. The prevailing antibiotic schedule consisted of the combination of ceftazidime plus amikacin, followed by piperacillin/tazobactam and carbapenems. Vancomycin was initially given in 174 cases (33.8%) whereas in another 101 ones (19.6%) this antibiotic was incorporated throughout the episode evolution. Antifungal therapy was administered on day 0 (n = 65) or during the follow-up of FNEs (n = 116). Seventy-one cases (13.8%) received antiviral therapy on day 0 including acyclovir (n = 45, 8.7%), antiretroviral drugs (n = 15, 2.9%) or other compounds (n = 11, 2.1%). Acyclovir recipients were those presenting cutaneous, mucosal or perianal herpes virus-like lesions. Antiviral treatment, at the onset of FNEs, was more prevalent in HIV-coinfected patients (26/51, 51%) if compared to the uninfected counterpart (45/460, 9,8%; p<0,001). In the ensuing days, 7 cases received acyclovir and another one antiretroviral treatments.

**Table 4:** Use of antimicrobials during febrile neutropenia episodes (n = 515).

**Initial antibiotic schedule other than vancomycin**	Ceftazidime + amikacin	171 (33.2%)
	Piperacillin / tazobactam	133 (25.8%)
	Carbapenems	60 (11.7%)
	Cefepime	40 (7.8%)
	Other combinations	111 (21.6%)
**Use of vancomycin**	At day 0	174 (33.8%)
	During evolution	101 (19.6%)
	Total	275 (53.4%)
**Use of antifungals**	At day 0	65 (12.6%)
	During evolution	116 (22.5%)
	Total	181 (35.1%)
**Use of antivirals**	At day 0	71 (13.8%)
	During evolution	8 (1.6%)
	Total	79 (15.3%)

### End of study

Hospital discharge was indicated in 346 FNEs, and in 77 episodes (14.9%), patients died during hospitalization. Thirteen episodes were referred to another center whereas, in the remaining 78 ones (15.1%), patients continued to be hospitalized for reasons other than febrile neutropenia. Median values for FNEs length, neutropenia duration, and fever persistence were 8 (5–13), 5 (3–8.25) and 4 (2–7) days, respectively.

### Variables associated with mortality at the end of study

The median neutropenia and fever duration, in days, were higher in fatal cases respect those who survived: 7.5 (3–13.25; 25–75% percentiles) vs. 5 (3–8), p = 0.003; and 6 (3–12.25) vs. 4 (2–6), p = 0.001, respectively.

Median neutrophil counts was 3 (0–280) for fatal cases, and 100 (0–350) for survivors (p = 0.033). Neutropenia lower than 50 cells/mm^3^ as a dichotomic variable was associated with an increased mortality (p = 0.023), this not being the case for those with neutrophils lower than 100 cells/mm^3^ (p = 0.35).

On crude analysis, MASCC risk index category was associated with mortality (Table 5). Other variables associated with mortality were: hospitalization at the onset of FNE, presence of CVC at presentation, treated infection before the onset FNEs, hypotension/tachycardia/ tachypnea/dehydration at the onset of FNE, initial use of vancomycin, unremitted fever at day 7 or unremitted neutropenia at day 14, as well as initial positive blood cultures, an ECOG score ≥ 3,or MASCC risk index <15, and neutrophils <50 cells/mm^3^ on day 0. Female gender, chemotherapy for a solid tumor cause of FNEs, and initial empirical treatment with ceftazidime plus amikacin showed a protective relationship with mortality on crude analysis. Variables that continued to show mortality-associated statistical significance, upon multivariate analysis, were the presence of dehydration and tachycardia, the existence of a treated infection before the onset FNEs, along with unremitted fever or neutropenia at days 7 and 14, respectively (Table 6).

**Table 5:** MASCC risk index at the onset of FNEs associated mortality.

	MASCC 0 to 11 (n = 199)	MASCC 12 to 15 (n = 182)	MASCC 16 to 20 (n = 110)	MASCC ≥21 (n = 24)	p*	contingency coefficient
**Mortality** **n (%)**	**47 (23.62%)**	**23 (12.64%)**	**6 (5.45%)**	**1 (4.17%)**	**<0.001**	**0,205**

MASCC: Multinational Association for Supportive Care in Cancer.

* Fisher´s exact test

**Table 6:** Variables associated with mortality during febrile neutropenia episodes.

Variable	Univariate analysis		Binary logistic regression analysis (n = 400)	
	p	Odds Ratio (CI 95%)	p	Adjusted Odds Ratio (CI 95%)
**Sex (women/men)** * (n = 515)	**0.048**	**0.60 (0.36–0.99)**	0.676	0.86 (0.43–1.73)
Age over 60 years (n = 515)	0.493	1.21 (0.70–2.07)		
Chronic obstructive pulmonary disease (n = 515)	0,758	0,56 (0.13–2.43)		
Post chemotherapy, solid tumor* (n = 515)	**0.008**	**0.35 (0.16–0.79)**	0.764	0.85 (0.30–2,41)
Post chemotherapy, oncohematological malignancies (n = 515)	0.710	1.10 (0.67–1.79)		
Hospitalized at the onset of FNE* (n = 512)	**0.002**	**2.25 (1.34–3.80)**	0.562	1.27 (0.57–2.83)
Previous radiotherapy (n = 514)	0.390	0.63 (0.22–1.82)		
Central venous catheter at the onset of FNE* (n = 514)	**0.001**	**2.40 (1.40–4.11)**	0.200	1.73 (0.75–4.01)
Bone marrow transplantation (n = 511)	1.000	0.70 (0.16–3.09)		
Previous episodes of febrile neutropenia (n = 515)	0.424	1.23 (0.74–2.4)		
Prior Use of Prophylactic antimicrobials (n = 513)	0.864	0.94 (0.44–1.99)		
Prior infections** (n = 514)	**<0.001**	**2.58 (1.51–4.42)**	**0.006**	**2.91 (1.36–6.22)**
Previous fungal invasive infection (n = 515)	0.270	1.68 (0.65–4.30)		
Hypotension at the onset of FNE* (n = 511)	**0.007**	**2.17 (1.23–3.82)**	0.147	1.87 (0.80–4.33)
Tachycardia at the onset of FNE** (n = 513)	**<0.001**	**2.79 (1.70–4.57)**	**0.048**	**2.21 (1.01–4.86)**
Oliguria at the onset of FNE (n = 512)	0.168	1.97 (0.81–4,81)		
Tachypnea at the onset of FNE* (n = 512)	**<0.001**	**2.74 (1.67–4.50)**	0.503	1.30 (0.60–2.81)
Dehydration at the onset of FNE** (n = 512)	**0.002**	**2.58 (1.40–4.75)**	**0.002**	**4.02 (1.64–9.87)**
Confirmed infection (n = 515)	0.608	1.15 (0.67–1.96)		
Use of vancomycin at the onset of FNE* (n = 515)	**0.002**	**2.16 (1.32–3.53)**	0.292	1.48 (0.71–3.09)
Unremitted fever at day 7** (n = 501)	**<0.001**	**2.94 (1.77–4.88)**	**0.046**	**2.07(1.01–4.24)**
Unremitted neutropenia at day 14** (n = 501)	**<0.001**	**3.50 (1.86–6.56)**	**0.008**	**3.28 (1.36–7.87)**
Initial positive blood culture* (n = 515)	**0.003**	**2.38 (1.32–4.30)**	0.150	1.80 (0.81–4.00)
Neutrophils <50 cells/mm3* (n = 464)	**0.023**	**1.82 (1.08–3.08)**	0.720	1.13 (0.57–2.25)
ECOG score ≥ 3 at the onset of FNE* (n = 472)	**<0.001**	**2.57 (1.53–4.33)**	0.289	1.47 (0.72–2.99)
Initial empiric therapy with ceftazidime-amikacin* (n = 507)	**0.001**	**0.36 (0.19–0.68)**	0.239	0.61 (0.27–1.39)
G-CSF non administration (n = 515)	0.436	1.22 (0.74–2.02)		
MASCC risk Index ≤ 15 at the onset of FNE* (n = 515)	**<0.001**	**4.08 (1.83–9.12)**	1.000	1.00 (0.31–3.20)

CI: confidence interval (95%); ECOG: Eastern Cooperative Oncology Group; FNE: febrile neutropenia episode; MASCC: Multinational Association for Supportive Care in Cancer. Variables yielding significant differences in crude analysis were entered into a binary logistic regression model for the multivariate approach.

*Statistical significance only on crude analysis

** Statistical significance upon employing multivariate analysis

After removing non-cancer patients, results did not deviate from the ones recorded in the whole sample, except for tachycardia, that lost statistical significance upon multivariate analysis (S1 Table).

## Discussion

According to study purposes, we herein report on the epidemiological, clinical, and microbiological features of patients with FNEs, their therapeutic management, and disease outcome. To our knowledge it constitutes the first report in terms of its multicentric and prospective nature in the region, enrolling a substantial number of FNEs.

Many published studies were based on retrospective data from patients enrolled in cancer clinical trials [4, 6, 9, 10, 18]. Some prospective studies in adults include only a few centers, with a smaller number of patients [19–23]. Other researchers, exclusively concentrate on post-chemotherapy neutropenia [13, 24, 25], only evaluate the first episode of febrile neutropenia [17], the ones occurring in the context of a new indication of chemotherapy [26], or they do not report further episodes occurring in the same patient [11].

Our approach of including all FNEs of any etiology, in 346 relatively young patients is closer to the real clinical scenario. In line with other studies [1, 8, 11, 19, 21, 27], most episodes (77%) were secondary to chemotherapy, although 106 cases of neutropenia of different etiologies were recorded. Also, more than half of patients were already hospitalized at the onset of FNEs, a situation known to confer a higher risk of complications and poor prognosis [11, 17, 25, 28], as did in the present series, although differences became insignificant upon multivariate analysis. Nearly 20% of cases were already receiving anti-infectious treatment at the onset of FNE, with this variable behaving as an independent mortality predictor.

According to the MASCC risk index [11], nearly all FNEs (95.3%) fell within the high-risk category, which is greater than values found in the studies from by Jin et al. (23.6%) [20] or Rabagliati et al. (41%) [22]. Reduced neutrophil counts at day 0, was found to be compatible with a poor prognosis of patients [5]. In the present series, a neutrophil count lower than 50 cells/mm^3^ was also related to a bad prognosis on crude analysis, although differences became statistical insignificant upon multivariate analysis.

While in only 20% of episodes fever had an unknown origin, in the remaining ones, an infectious process was detected based on clinical (46%), microbiological (11%) or combined (23%) criteria. Such amount of recognized infections is higher than findings from other series, although more recent studies show values above figures recorded in earlier studies [8, 21, 27, 29]. The increased presence of infections may be due to the availability of better diagnostic methods, although it cannot be ruled out that single clinical criteria may have led to an overestimation of it. Overall, the distribution of affected sites agrees with findings from other studies of FNEs, that is pneumonia, the involvement of the gastrointestinal tract, skin and soft tissues, and the oral cavity [1, 5, 8, 21]. As well as in other reports, bacteremia was present between 10 and 37% of blood samples [5, 8, 24], 15% of present FNEs had positive blood cultures. In a study carried out in Buenos Aires [19], 36.9% of blood cultures yielded positive results, like two Chilean studies reporting 30.5% and 31.4% positivity [21, 22]. Our lower bacteremia may be due to that more than 30% of these patients were under antimicrobial treatment before the onset of FNEs, either for prophylactic reasons or the existence of a prior infection. We only found an insignificant trend for Gram-positive bacteriemia in cases under antibiotic prophylaxis, probably due to its limited use in our patient series.

Following the Gram-negative to Gram-positive shift in pathogen predominance seen worldwide during the 1980s, Gram-positive bacteria became predominant in most industrialized regions, with less developed countries still showing a Gram-negative bacteria predominance, possibly related to lower antibiotic prophylaxis [30–35]. However, in the last decade, most centers report similar Gram-positive and Gram-negative bacteremia rates in patients with FNEs [6, 36]. In agreement with studies from the region [21] or Southeast Asia [20], our series also revealed a slight predominance of Gram-negative bacteria (52.8%). As seen in former reports [1, 5, 20–22, 27, 37, 38] we also found a predominance of *E*. *coli*, *K*. *pneumoniae* and pseudomonas species. Within Gram-positive bacteria, methicillin-susceptible *Staphylococcus aureus* and methicillin-resistant *Staphylococcus aureus* followed by coagulase-negative staphylococci were the prevailing forms. The latter one was also found to predominate among Gram-positive cocci [20–22, 37].

The fact that G-CSF was indicated in 66% of the FNEs suggests some overuse, as current guidelines mostly recommend its administration before the establishment of FNEs in patients at high risk [8, 36, 39–41]. However, recent guideline recommendations indicate that G-CSF should be considered in patients with fever and neutropenia who are at high risk for infection-associated complications or have prognostic factors predictive of poor clinical outcomes [40].

The commonest antibiotic schedule employed in this patient series consisted of ceftazidime plus amikacin, followed by piperacillin/tazobactam, or carbapenems and cefepime as monotherapy. The latest guidelines from the Argentine Society of Infectious Diseases [5] and the Infectious Diseases Society of America [8] and meta-analytical approaches [42] regard ceftazidime plus amikacin as a second choice in favor of the abovementioned alternatives. Most cases from the present studies were included before the availability of these guidelines [5, 8] when ceftazidime plus amikacin were recommended as the initial empirical treatment [3, 43, 44]. Nevertheless, since evidence is not conclusive in this regard [45], these therapeutic schedules continue to be adequate for the management of FNEs, with other recent guidelines still including ceftazidime as first-line therapy [36, 46, 47]. In our hands, although initial treatment with ceftazidime plus amikacin was associated with lower mortality -probably because such treatment was not of first choice for severe patients-, further multivariate analysis revealed no differences in mortality depending on the initial antimicrobial therapy.

The fact that vancomycin was indicated in more than half of FNEs raises the possibility of some overuse. Vancomycin has precise indications in this kind of patients [5, 8], for which institutional surveillance is currently being implemented to establish more accurate handling of it. In the same sense, the use of antifungal therapy (35.1% of episodes) seems high, given the lower amount of cases with confirmed invasive mycosis. The fact that antifungal therapy does not require mycological confirmation [5, 8, 48, 49], and patients presented prolonged and severe neutropenia mostly linked to oncohematological malignancies, may have accounted for such indication.

In line with other studies [3, 41], the mean recovery time of neutrophils situated between 6 and 8 days. Results also revealed that the longer the time for neutrophil recovering (over 14 days in our series) the poorer the prognosis, as seen in former reports [37, 43].

On admission, patients fit well with the high-risk category (95.3%), showing a mortality rate (14.9%) like the one recorded in a study of comparable risk [22]. Mortality rate increase with the MASCC prognostic index, in agreement with other studies showing mortality as low as 3% if the MASCC score was >21, further reaching to 36% provided the MASCC score was <15 [10]. As stated, patients were at high risk of complications, i.e., increased MASCC risk index and ECOG scores, low neutrophil counts, prolonged FNEs, many already hospitalized patients with previous FNEs, Gram-negative bacteremia, as well as a high occurrence of hematological neoplasms, and lung involvement. According to multivariate analysis, mortality-associated variables were: unremitted fever at day 7, unremitted neutropenia at day 14, tachycardia, dehydration, and previous infections. The lost of MASCC statistical significance following multivariate analysis may be related to the fact that some of the adjusting variables were also employed for the score construction. As stated, analysis among oncologic patients (the MASCC score was originally developed for them), revealed a quite similar pattern of mortality association. Except for tachycardia which was only associated with mortality in the multivariate analysis from the whole patient sample. The lower number of FNEs among oncologic patients may account for such lost of statistical association.

The study by Gómez Roca et al. [19] identified the presence of comorbidities, tachypnea, systolic hypotension, and a clinical infectious process as factors of poor prognosis.

This type of observational study has limitations. For instance, there existed variations in causes of febrile neutropenia, and features of centers in charge of patients. Management was decided at each center according to its usual practice, likely contributing to some therapeutic dissimilarities. In the same sense, surveillance for multidrug-resistant bacteria was not usually carried out in many centers, for which data regarding this issue is lacking, as did information about *Clostridium difficile* toxin. Also, there was no information on lifestyle parameters, primary healthcare data, or socioeconomic data, emerging as potential confounding factors.

Against such constraints, our study did collect data about underlying diseases which may influence study endpoints and revealed a consistent and relatively high association, likely to reduce the risk of unknown confounders. These facts, along with the proper representativeness of centers allowing to get an informative number of cases strengthen study validity serving to expand present knowledge on the clinical and microbiological features of patients with febrile neutropenia in Argentina. Present kind of observational data also help to identify associations between exposures and outcomes, and the strength of such associations, providing useful information for clinical decisions and better management of FNEs in the region.

## Supporting information

S1 Table Variables associated with mortality during febrile neutropenia episodes excluding non-cancer patients. (DOCX)

S1 File Five hundred and fifteen febrile neutropenia episodes in Argentina during a 5-year period database. (SAV)

## References

[1] Crawford J, Dale DC, Lyman GH. Chemotherapy-induced neutropenia: risks, consequences, and new directions for its management. Cancer. 2004; 100: 228–37. 10.1002/cncr.11882 14716755

[2] Jacobson MA, Liu RC, Davies D, Cohen PT. Human immunodeficiency virus disease-related neutropenia and the risk of hospitalization for bacterial infection. Arch Intern Med. 1997; 157: 1825–31. 9290541

[3] Hughes WT, Armstrong D, Bodey GP, Bow EJ, Brown AE, Calandra T, et al 2002 guidelines for the use of antimicrobial agents in neutropenic patients with cancer. Clin Infect Dis. 2002; 34: 730–51. 10.1086/339215 11850858

[4] Kurtin S. Myeloid toxicity of cancer treatment. J Adv Pract Oncol. 2012; 3: 209–24. 25031949 PMC4093344

[5] Sociedad Argentina De Infectologia. Guías de recomendaciones sobre diagnóstico, tratamiento y prevención de infecciones en pacientes con cáncer 2013. Rev. argent. microbiol. [online]. 2014;46, suppl.1:7–144.24721268

[6] Viscoli C, Varnier O, Machetti M. Infections in patients with febrile neutropenia: epidemiology, microbiology, and risk stratification. Clin Infect Dis. 2005; 40 Suppl 4: S240–5. 15768329 10.1086/427329

[7] Kruger P, Saffarzadeh M, Weber AN, Rieber N, Radsak M, von Bernuth H, et al Neutrophils: Between host defence, immune modulation, and tissue injury. PLoS Pathog. 2015; 11: e1004651 10.1371/journal.ppat.1004651 25764063 PMC4357453

[8] Freifeld AG, Bow EJ, Sepkowitz KA, Boeckh MJ, Ito JI, Mullen CA, et al Infectious Diseases Society of America. Clinical practice guideline for the use of antimicrobial agents in neutropenic patients with cancer: 2010 update by the infectious diseases society of America. Clin Infect Dis. 2011; 52: e56–93. 10.1093/cid/cir073 21258094

[9] Viscoli C; EORTC International Antimicrobial Therapy Group. Management of infection in cancer patients. Studies of the EORTC International Antimicrobial Therapy Group (IATG). Eur J Cancer. 2002; 38 Suppl 4: S82–7. 11858971 10.1016/s0959-8049(01)00461-0

[10] de Naurois J, Novitzky-Basso I, Gill MJ, Marti FM, Cullen MH, Roila F; ESMO Guidelines Working Group. Management of febrile neutropenia: ESMO Clinical Practice Guidelines. Ann Oncol. 2010; 21 Suppl 5: v252–6. 20555092 10.1093/annonc/mdq196

[11] Klastersky J, Paesmans M, Rubenstein EB, Boyer M, Elting L, Feld R, et al The Multinational Association for Supportive Care in Cancer risk index: A multinational scoring system for identifying low-risk febrile neutropenic cancer patients. J Clin Oncol. 2000; 18: 3038–51. 10.1200/JCO.2000.18.16.3038 10944139

[12] Oken MM, Creech RH, Tormey DC, Horton J, Davis TE, McFadden ET, et al Toxicity and response criteria of the Eastern Cooperative Oncology Group. Am J Clin Oncol. 1982; 5: 649–55. 7165009

[13] Sørensen JB, Klee M, Palshof T, Hansen HH. Performance status assessment in cancer patients. An inter-observer variability study. Br J Cancer. 1993; 67: 773–5. 10.1038/bjc.1993.140 8471434 PMC1968363

[14] Innes H, Lim SL, Hall A, Chan SY, Bhalla N, Marshall E. Management of febrile neutropenia in solid tumours and lymphomas using the Multinational Association for Supportive Care in Cancer (MASCC) risk index: feasibility and safety in routine clinical practice. Support Care Cancer. 2008; 16: 485–91. 10.1007/s00520-007-0334-8 17899215

[15] Klastersky J, Paesmans M. The Multinational Association for Supportive Care in Cancer (MASCC) risk index score: 10 years of use for identifying low-risk febrile neutropenic cancer patients. Support Care Cancer. 2013; 21: 1487–95 10.1007/s00520-013-1758-y 23443617

[16] Uys A, Rapoport BL, Anderson R. Febrile neutropenia: a prospective study to validate the Multinational Association of Supportive Care of Cancer (MASCC) risk-index score. Support Care Cancer. 2004; 12: 555–60. 10.1007/s00520-004-0614-5 15197637

[17] Carmona-Bayonas A, Gómez J, González-Billalabeitia E, Canteras M, Navarrete A, Gonzálvez ML, et al Prognostic evaluation of febrile neutropenia in apparently stable adult cancer patients. Br J Cancer. 2011; 105: 612–7. 10.1038/bjc.2011.284 21811253 PMC3188929

[18] Chan A, Verma S, Loibl S, Crawford J, Choi MR, Dreiling L, et al Reporting of myelotoxicity associated with emerging regimens for the treatment of selected solid tumors. Crit Rev Oncol Hematol. 2012; 81:136–50. 10.1016/j.critrevonc.2011.03.003 21507676

[19] Gómez Roca C, Rivero M, Hugo K, Novillo A, Marta Lapadula M, Recondo G, et al Factores de mal pronóstico en pacientes internados con neutropenia al inicio del episodio febril. M. Medicina (B Aires). 2006; 66:385–91. 17137166

[20] Jin J, Lee YM, Ding Y, Koh LP, Lim SE, Lim R, et al Prospective audit of febrile neutropenia management at a tertiary university hospital in Singapore. Ann Acad Med Singapore. 2010; 39:453–9. 20625621

[21] Rabagliati B R, Fuentes L G, Orellana U E, Oporto C J, Domínguez M I, Benítez G R, et al Etiología de episodios de neutropenia febril en pacientes adultos con cáncer hematológico y de órganos sólidos en el Hospital Clínico Universidad Católica, Santiago-Chile. Rev Chilena Infectol. 2009; 26:106–13. 19621141

[22] Rabagliati R, Bertín P, Cerón I, Rojas H, Domínguez I, Vera Á, et al Epidemiología de neutropenia febril en pacientes adultos con leucemia aguda y linfoma. Estudio de cohorte en hospitales público y privado de Santiago, Chile. Cruz R. Rev Chilena Infectol. 2014; 31:721–8. 10.4067/S0716-10182014000600013 25679930

[23] Sacar S, Hacioglu SK, Keskin A, Turgut H. Evaluation of febrile neutropenic attacks in a tertiary care medical center in Turkey. J Infect Dev Ctries. 2008; 2:359–63. 19745503 10.3855/jidc.197

[24] Klastersky J, Ameye L, Maertens J, Georgala A, Muanza F, Aoun M, et. al Bacteraemia in febrile neutropenic cancer patients. Int J Antimicrob Agents. 2007; 30 Suppl 1: S51–9. 17689933 10.1016/j.ijantimicag.2007.06.012

[25] Talcott JA, Siegel RD, Finberg R, Goldman L. Risk assessment in cancer patients with fever and neutropenia: a prospective, two-center validation of a prediction rule. J Clin Oncol. 1992; 10: 316–22. 10.1200/JCO.1992.10.2.316 1732432

[26] Lyman GH, Kuderer NM, Crawford J, Wolff DA, Culakova E, Poniewierski MS, et al Predicting individual risk of neutropenic complications in patients receiving cancer chemotherapy. Cancer. 2011; 117: 1917–27. 10.1002/cncr.25691 21509769 PMC3640637

[27] Kamana M, Escalante C, Mullen CA, Frisbee-Hume S, Rolston KV. Bacterial infections in low-risk, febrile neutropenic patients. Cancer. 2005; 104:422–6. 10.1002/cncr.21144 15937905

[28] Talcott JA, Finberg R, Mayer RJ, Goldman L. The medical course of cancer patients with fever and neutropenia. Clinical identification of a low-risk subgroup at presentation. Arch Intern Med. 1988; 148: 2561–8. 3196123

[29] Pizzo PA, Robichaud KJ, Gill FA, Witebsky FG. Empiric antibiotic and antifungal therapy for cancer patients with prolonged fever and granulocytopenia. Am J Med. 1982; 72: 101–11. 10.1016/0002-9343(82)90594-0 7058815

[30] Kanafani ZA, Dakdouki GK, El-Chammas KI, Eid S, Araj GF, Kanj SS. Bloodstream infections in febrile neutropenic patients at a tertiary care center in Lebanon: a view of the past decade. Int J Infect Dis. 2007;11: 450–3 10.1016/j.ijid.2006.12.008 17337226

[31] Gafter-Gvili A, Fraser A, Paul M, Vidal L, Lawrie TA, van de Wetering MD, et al Antibiotic prophylaxis for bacterial infections in afebrile neutropenic patients following chemotherapy. Cochrane Database Syst Rev. 2012; 1: CD004386 10.1002/14651858.CD004386.pub3 22258955 PMC4170789

[32] Gafter-Gvili A, Fraser A, Paul M, Leibovici L. Meta-analysis: antibiotic prophylaxis reduces mortality in neutropenic patients. Ann Intern Med. 2005; 42: 979–95. 10.7326/0003-4819-142-12_part_1-200506210-00008 15968013

[33] Gafter-Gvili A, Fraser A, Paul M, Van de Wetering M, Kremer L, Leibovici L. Antibiotic prophylaxis for bacterial infections in afebrile neutropenic patients following chemotherapy (Review). Cochrane Database Syst Rev. 2005;4:CD004386. 10.1002/14651858.CD004386.pub2 16235360

[34] Gafter-Gvili A, Paul M, Fraser A, Leibovici L. Effect of quinolone prophylaxis in afebrile neutropenic patients on microbial resistance: systematic review and meta-analysis. J Antimicrob Chemother. 2007; 59: 5–22. 10.1093/jac/dkl425 17077101

[35] Elting LS, Rubenstein EB, Rolston KV, Bodey GP. Outcomes of bacteremia in patients with cancer and neutropenia: observations from two decades of epidemiological and clinical trials. Clin Infect Dis. 1997; 25: 247–59. 10.1086/514550 9332520

[36] Klastersky J, de Naurois J, Rolston K, Rapoport B, Maschmeyer G, Aapro M, et. Al ESMO Guidelines Committee. Management of febrile neutropaenia: ESMO Clinical Practice Guidelines. Ann Oncol. 2016; 27(suppl 5):v111–v118. 10.1093/annonc/mdw325 27664247

[37] Feld R. Bloodstream infections in cancer patients with febrile neutropenia. Int J Antimicrob Agents. 2008; 32 Suppl 1: S30–3. 18778919 10.1016/j.ijantimicag.2008.06.017

[38] Villafuerte-Gutierrez P, Villalon L, Losa JE, Henriquez-Camacho C. Treatment of febrile neutropenia and prophylaxis in hematologic malignancies: a critical review and update. Adv Hematol. 2014; 986938 10.1155/2014/986938 25525436 PMC4265549

[39] Smith TJ, Khatcheressian J, Lyman GH, Ozer H, Armitage JO, Balducci L, et. al 2006 update of recommendations for the use of white blood cell growth factors: an evidence-based clinical practice guideline. J Clin Oncol. 2006;24:3187–205. 10.1200/JCO.2006.06.4451 16682719

[40] Smith T. J., Bohlke K., Lyman G. H., Carson K. R., Crawford J., Cross S. J. et al American Society of Clinical Oncology. Recommendations for the Use of WBC Growth Factors: American Society of Clinical Oncology Clinical Practice Guideline Update. J Clin Oncol. 2015;33:3199–212. 10.1200/JCO.2015.62.3488 26169616

[41] Ozer H, Armitage JO, Bennett CL, Crawford J, Demetri GD, Pizzo PA, et al American Society of Clinical Oncology. 2000 update of recommendations for the use of hematopoietic colony-stimulating factors: evidence-based, clinical practice guidelines. American Society of Clinical Oncology Growth Factors Expert Panel. J Clin Oncol. 2000; 18: 3558–85. 10.1200/JCO.2000.18.20.3558 11032599

[42] Horita N, Shibata Y, Watanabe H, Namkoong H, Kaneko T. Comparison of antipseudomonal β-lactams for febrile neutropenia empiric therapy: systematic review and network meta-analysis. Clin Microbiol Infect. 2017;23:723–729. 10.1016/j.cmi.2017.03.024 28377312

[43] Cozzi JA. Guías de recomendaciones sobre diagnóstico, tratamiento y prevención de infecciones en pacientes con cáncer. Consenso de la Sociedad Argentina de Infectología, Comisión de Infecciones en el Paciente Neoplásico y Trasplantado de Médula Ósea, 2008.

[44] Baskaran ND, Gan GG, Adeeba K, Sam IC. Bacteremia in patients with febrile neutropenia after chemotherapy at a university medical center in Malaysia. Int J Infect Dis 2007; 6: 513–17. 10.1016/j.ijid.2007.02.002 17459753

[45] Anoshirvani AA, Zarinfar N, Rafiee M, Zamani Z. Effect of Combination Therapy of Ceftazidime/Amikacin and Monotherapy with Imipenem on the Treatment of Fever and Neutropenia in Patients with Cancers. Open Access Maced J Med Sci. 2018;6:1423–1430 10.3889/oamjms.2018.310 30159069 PMC6108819

[46] Heinz WJ, Buchheidt D, Christopeit M, von Lilienfeld-Toal M, Cornely OA, Einsele H, et al Diagnosis and empirical treatment of fever of unknown origin (FUO) in adult neutropenic patients: guidelines of the Infectious Diseases Working Party (AGIHO) of the German Society of Hematology and Medical Oncology (DGHO). Ann Hematol. 2017;96:1775–1792. 10.1007/s00277-017-3098-3 28856437 PMC5645428

[47] Schmidt-Hieber M, Teschner D, Maschmeyer G, Schalk E. Management of febrile neutropenia in special consideration of the role of antimicrobial de-escalation. Expert Rev Anti Infect Ther. 2019 1 28 10.1080/14787210.2019.1573670 30686067

[48] Prentice HG, Kibbler CC, Prentice AG. Towards a targeted, risk-based, antifungal strategy in neutropenic patients. Br J Haematol. 2000; 110: 273–84. 10.1046/j.1365-2141.2000.02014.x 10971382

[49] Corey L, Boeckh M. Persistent fever in patients with neutropenia. N Engl J Med. 2002; 346: 222–4. 10.1056/NEJM200201243460402 11807145

