# Supplementary material for: A multicenter prospective study of 515 febrile neutropenia episodes in Argentina during a 5-year period
Source: PLoS One. 2019 Oct 31;14(10):e0224299. doi: 10.1371/journal.pone.0224299 (PMC6822758; doi:10.1371/journal.pone.0224299)
Supplement: S1 Table — (DOCX) [file pone.0224299.s001.docx]

**S1 Table. Variables associated with mortality during febrile neutropenia episodes excluding non-cancer patients.**

| Variable | Univariate analysis | | Binary logistic regression analysis (n=341) | |  |
| --- | --- | --- | --- | --- | --- |
|  | P | Odds Ratio (CI 95%) | p | Adjusted Odds Ratio (CI 95%) | |
| Sex (women/men) * (n= 431) | **0.036** | **0.55 (0.31-0.97)** | 0.461 | 0.74 (0.33-1.65) | |
| Age over 60 years (n= 431) | 0.470 | 1.24 (0.69-2.23) |  |  | |
| Chronic obstructive pulmonary disease (n=431) | 0.298 | 0.36 (0.5-2.72) |  |  | |
| Post chemotherapy, solid tumor* (n= 431) | **0.009** | **0,35 (0.15-0.79)** | 0.938 | 0.96 (0.32-2.87) | |
| Post chemotherapy, oncohematological malignancies (n= 431) | 0.473 | 1.24 (0.69-2.23) |  |  | |
| Hospitalized at the onset of FNE* (n= 429) | **0.002** | **2.50 (1.49-4.48)** | 0.371 | 1.53 (0.61-3.85) | |
| Previous radiotherapy (n= 430) | 0.451 | 0.66 (0.23-1.94) |  |  | |
| Central venous catheter at the onset of FNE* (n= 430) | **0.010** | **2.12 (1.18-3.80)** | 0.316 | 1.61 (0.63-4.10) | |
| Bone marrow transplantation (n= 429) | 0.756 | 0.72 (1.16-3.29) |  |  | |
| Previous episodes of febrile neutropenia (n= 431) | 0.312 | 1.32 (0.77-2.30) |  |  | |
| Prior Use of Prophylactic antimicrobials (n= 429) | 0.427 | 0.70 (0.29-1.70) |  |  | |
| Prior infections** (n= 430) | **0.001** | **2.79 (1.49-5.23)** | **0.036** | **2.66 (1.07-6.62)** | |
| Previous fungal invasive infection (n= 431) | 0.736 | 1.18 (0.33-4.19) |  |  | |
| Hypotension at the onset of FNE* (n= 427) | **0.013** | **2.15 (1.16-3.99)** | 0.262 | 1.74 (0.66-4.58) | |
| Tachycardia at the onset of FNE* (n= 429) | **<0.001** | **2.87 (1.67-4.95)** | 0.125 | 2.01 (0.82-4.94) | |
| Oliguria at the onset of FNE (n= 428) | 0.088 | 2.30(0.86-6.11) |  |  | |
| Tachypnea at the onset of FNE* (n= 428) | **<0.001** | **3.10 (1.79-5.36)** | 0.232 | 1.72 (0.71-4.18) | |
| Dehydration at the onset of FNE** (n= 428) | **0.008** | **2.46 (1.24-4.86)** | **0.005** | **4.61 (1.59-13.36)** | |
| Confirmed infection (n= 431) | 0.944 | 0.98 (0.56-1.73) |  |  | |
| Use of vancomycin at the onset of FNE* (n= 431) | **0.030** | **1,81 (1.06-3.11)** | 0.817 | 1..1 (0.48-2.57) | |
| Unremitted fever at day 7** (n= 424) | **<0.001** | **3.90 (2.22-6.84)** | **0.023** | **2.54 (1.14-5.64)** | |
| Unremitted neutropenia at day 14** (n= 424) | **<0.001** | **3.80 (1.93-7.51)** | **0.010** | **3.55 (1.36-9.28)** | |
| Initial positive blood culture* (n= 431) | **0.011** | **2.27 (1.19-4.31)** | 0.165 | 1.86 (0.77-4.48) | |
| Neutrophils <50 cells/mm3* (n= 390) | **0.011** | **2.13 (1.18-3.85)** | 0.180 | 1.72 (0.78-3.77) | |
| ECOG score ≥ 3 at the onset of FNE* (n= 391) | **0.009** | **2.15-1.20-3.86)** | 0.566 | 1.26 (0.57-2.78) | |
| Initial empiric therapy with ceftazidime-amikacin* (n= 425) | **0.003** | **0.36 (0.18-0.72)** | 0.221 | 0.56 (0.22-1.42) | |
| G-CSF non administration (n= 431) | 0.907 | 1.59 (0.92-2.74) |  |  | |
| MASCC risk Index ≤ 15 at the onset of FNE* (n= 431) | **0.001** | **3.94 (1.65-9.43)** | 0.927 | 0.94 (0.25-3.59) | |

CI: confidence interval (95%); ECOG: Eastern Cooperative Oncology Group; FNE: febrile neutropenia episode; MASCC: Multinational Association for Supportive Care in Cancer. Variables yielding significant differences in crude analysis were entered into a binary logistic regression model for the multivariate approach.

***Statistical significance only on crude analysis**

**** Statistical significance upon employing multivariate analysis**
